# Replication Study of Polymorphisms Associated With Brain Arteriovenous Malformation in a Population From South of Brazil

**DOI:** 10.7759/cureus.508

**Published:** 2016-02-24

**Authors:** André Cerutti Franciscatto, Fernanda S Ludwig, Ursula S Matte, Simone Mota, Marco A Stefani

**Affiliations:** 1 Neurosurgery, Hospital Mãe de Deus - Porto Alegre - Brazil; Hospital Dom João Becker - Gravataí - Brazil; 2 Hospital de Clínicas de Porto Alegre, Universidade Federal do Rio Grande do Sul

**Keywords:** brain arteriovenous malformation, single nucleotide polymorphisms, apolipoprotein e, activine-like kinase 1, endoglin, tumor necrosis factor alpha, interleukin 1 beta, interleukin 6

## Abstract

**Introduction:**

The aim of this study was to reproducibly determine if any of the polymorphisms were associated with the susceptibility to brain arteriovenous malformations (BAVM) or the risk of intracranial hemorrhage (ICH) presentation.

**Methods:**

We recruited 63 BAVM patients and 96 controls. The polymorphisms selected for evaluation were apolipoprotein E (APOE), tumor necrosis factor alpha (TNF 238G>A - rs361525), interleukin 1 beta (IL1B 511C>T - rs16944 and IL1B -31T>C - rs1143627), activin-like kinase 1 (ACVRL1 IVS3-35A>G - rs2071219), endoglin (ENG 207G>A - rs11545664), and interleukin 6 (IL6 174G>C - rs1800795).

**Results:**

In the single analysis, we observed statistically signiﬁcant differences in the allele distributions for IL1B -31T>C (rs1143627) between the BAVM patients and control subjects (P = 0.02). There was a trend toward significance for the association between the IL1B 511C>T (rs16944) allele and BAVM risk (P = 0.07). In further logistic regression analysis, no polymorphism was significantly associated with the risk of BAVM. No polymorphisms were associated with hemorrhage presentation according to both single and multivariable analyses.

**Conclusions:**

In our sample from a south Brazil population, we found no association between the risks of BAVM and ICH presentation with any of the selected polymorphisms.

## Introduction

Brain arteriovenous malformations (BAVM) are a cause of intracranial hemorrhage (ICH). The basic morphology of BAVM consists of a tangle of abnormal and dilated channels with intervening gliosis, called the nidus, that directly shunts blood circulation between the arteries and veins without a true capillary bed [1]. The prevention of BAVM bleeding or rebleeding is the aim of the treatment. Improving the genetic knowledge of BAVMs could lead to the development of new forms of treatments and future prevention therapies.

Single nucleotide polymorphisms (SNPs), which are common in the human genome, have been shown to play an important role in the prediction and diagnosis of diseases [2].

We genotyped seven polymorphisms in genes associated with the inflammatory response and angiogenic pathways based on results and biological hypothesis of previous studies. The aim of this study was to reproducibly determine if any of the polymorphisms were associated with the susceptibility to BAVM or the risk of hemorrhagic presentation.

## Materials and methods

This case-control study on genetic association used the STrengthening the REporting of Genetic Associations (STREGA) statement for elaborating, conducting, and reporting research [3].

A total of 63 patients with BAVM were recruited from the Department of Neurosurgery of Hospital de Clínicas de Porto Alegre (HCPA), Brazil, as a part of our larger HCPA BAVM registry.

In addition, 96 healthy volunteers served as the controls. The inclusion criteria for the controls were as follows: (I) aged 18 years to 65 years; (II) the absence of a history of severe brain trauma; and (III) the absence of a family history of cerebrovascular diseases or intracranial tumors. All of the patients and controls were assessed for age, sex, and ethnicity (self-reported). The BAVM patients were evaluated for the Spetzler-Martin grade and clinical presentation. Intracranial blood on previous computed tomography or magnetic resonance imaging and a history of intracranial hemorrhage (ICH) were used to define BAVM presentation, and these patients were grouped as ‘ruptured’. The patients who did not contain evidence of bleeding but presented with seizure, focal ischemic deficit, headache, and apparently unrelated symptoms or were asymptomatic were grouped as ‘unruptured’.

Each participant provided informed consent, and the study was approved by the Human Subjects Review Committee of HCPA.

The genes selected for evaluation were apolipoprotein E (APOE), tumor necrosis factor alpha (TNF), interleukin 1 beta (IL1B), activin-like kinase 1 (ACVRL1), endoglin (ENG), and interleukin 6 (IL6).

Fasting venous blood was collected, and deoxyribonucleic acid was harvested as previously described [4]. The blood samples were stored at –80ºC until use. TaqMan® SNP Genotyping (Life Technologies, Carlsbad, CA, USA) was used to study the SNPs TNF -238G>A (rs361525), IL1B -511C>T (rs16944), IL1B -31T>C (rs1143627), ACVRL1 IVS3-35A>G (rs2071219), ENG 207G>A (rs11545664), and IL6 -174G>C (rs1800795), and direct Sanger sequencing was used for genotyping APOE.

The statistical analysis was performed using the Statistical Package for the Social Sciences version 20.0 (SPSS, Chicago, IL, USA).

Hardy-Weinberg equilibrium (HWE) was tested with the Pearson’s chi-squared (χ²) test. If HWE was present in the controls, we used both the dominant and multiplicative models for the minor allele to calculate the Odds Ratio (OR) and 95% confidence intervals (95% CI) for BAVM risk. If HWE was not present in the control group, we calculated OR based only on a dominant model for the minor allele.

The categorical data and rates were compared with the χ² -test. The Fisher’s exact probability test was used if the distributional assumptions or an expected value of at least five observations in each cell were not achieved. Continuous variables (e.g., age at presentation) were analyzed with a t-test. The Mann-Whitney test was used if a non-normal distribution was present.

A multivariate regression model was performed to control for gender, age, and ethnicity for BAVM risk. We used the Spetzler-Martin grade in multivariable analysis for the risk of hemorrhage presentation.

The overall value of P < 0.05 was considered statistically significant.

## Results

The demographics of the groups are shown in Table 1.

**Table 1:** Demographics of the subjects and genotypes of selected polymorphisms. BAVM, brain arteriovenous malformation; SD, standard deviation. Gender, ethnicity, and full genotype frequencies in patients and controls were compared using a 2-sided χ²-test. The ages (at research entry) of the patients and controls were compared using a t-test.

Group	BAVM cases (n=62)	Controls (n=96)	P-Value
Gender			0.18
Male (%)	30 (48)	57 (59)	
Female (%)	32 (52)	39 (41)	
Age (Years)			0.27
Mean±SD	41.4 ± 18.2	37.1 ± 12.5	
Ethnicity			0.75
Caucasian (%)	50 (80.6)	82 (85.4)	
Afro Brazilians (%)	4 (6.5)	6 (6.3)	
Admixed (%)	8 (12.9)	8 (8.3)	
APOE			0.22
E2 +	6 (9.7)	16 (16.7)	
E2 -	56 (90.3)	80 (83.3)	
TNF (rs361525)			0.63
AA	0 (0)	0 (0)	
AG	8 (12.9)	10 (10.4)	
GG	54 (87.1)	86 (89.6)	
IL1B (rs16944)			0.11
AA	4 (6.5)	17 (17.7)	
AG	32 (51.6)	47 (49)	
GG	26 (41.9)	32 (33.3)	
IL1B (rs1143627)			0.07
GG	10 (16.1)	23 (24)	
AG	26 (41.9)	49 (51)	
AA	26 (41.9)	24 (25)	
ACVRL1 (rs2071219)			0.66
GG	8 (12.9)	11 (11.5)	
AG	31 (0.5)	55 (57.2)	
AA	23 (37.1)	30 (31.3)	
ENG (rs11545664)			0.35
TT	0 (0)	3 (3.1)	
CT	13 (21)	21 (21.9)	
CC	49 (79)	72 (75)	
IL6 (rs1800795)			0.97
CC	6 (9.7)	10 (10.4)	
CG	25 (40.3)	37 (38.5)	
GG	31 (0.5)	49 (51)	

There were no significant differences in age, ethnicity or gender between the patients with BAVM and the controls. Aside from gender frequency, we found no differences in demographic or BAVM characteristics between the patients with or without ICH presentation (Table 2).

**Table 2:** Demographic and BAVM characteristics in ruptured and unruptured BAVM patients. ICH, Intracranial Hemorrhage; SD, standard deviation; Gender and ethnicity frequencies in the patients and controls were compared using a 2-sided χ²-test. The age at diagnosis in patients with and without ICH was compared using a t-test.

	ICH (n=38)	Unruptured (n=24)	Total (n=62)	P
Gender				0.02
Male (%)	14 (36.8)	16 (66.67)	30 (48)	
Female (%)	24 (63.2)	8 (33.33)	32 (52)	
Age at diagnosis (year)				0.3
Mean age±SD	32.7 ± 18	40.2 ± 15.8		
Ethnicity				0.84
Caucasian (%)	30 (78.9)	20 (83.3)	50 (80.6)	
Afro Brazilians (%)	3 (7.9)	1 (4.2)	4 (6.5)	
Admixed (%)	5 (13.2)	3 (12.5)	8 (12.9)	
Spetzler-Martin grade				0.19
I	3	6	9	
II	8	7	15	
III	18	9	27	
IV	7	1	8	
V	2	1	3	

The genotype frequency distributions of all seven polymorphisms were consistent with HWE both in the cases and controls (Table 3). All SNPs in our study population had a minor allele frequency greater than 5%.

In the single analysis, we observed statistically signiﬁcant differences in the allele distributions for IL1B -31T>C (rs1143627) between the BAVM patients and control subjects (P = 0.02). There was a trend towards significance for the association of IL1B -511C>T (rs16944) with BAVM risk (P = 0.07). In a logistic regression analysis that was adjusted for the Caucasian ethnicity, age, and the male gender, there were no significant associations between the polymorphisms and the risk of BAVM (Table 3).

**Table 3:** The association of studied polymorphisms with brain arteriovenous malformation. OR, odds ratio calculated both in dominant and multiplicative models for the minor allele; CI, confidence interval; The single analysis calculated P values were based on 2-sided χ²-tests. Logistic regression was used for multivariable analysis controlling for Caucasian ethnicity, age and, male sex.

	Single Analysis		Multivariable Analysis			
Risk Factor	OR	95% CI	P	OR	95% CI	P
APOE						
E2 carriers	0.54	0.2-1.45	0.22	0.93	0.3-2.89	0.89
ACVRL1 (rs2071219)						
Any G vs AA	0.77	0.39-1.51	0.45	0.99	0.4-2.44	0.98
G vs A	0.91	0.57-1.45	0.69	1.18	0.62-2.27	0.62
ENG (rs11545664)						
Any T vs CC	0.8	0.37-1.71	0.56	0.42	0.12-1.47	0.18
T vs C	0.72	0.35-1.45	0.35	0.37	0.11-1.23	0.1
TNF (rs361525)						
Any A vs GG	1.27	0.47-3.43	0.63	2.41	0.71-8.23	0.16
A vs G	1.26	0.48-3.27	0.64	2.6	0.8-8.55	0.12
IL1B (rs16944)						
Any A vs GG	0.69	0.36-1.34	0.27	3.35	0.37-30.56	0.29
A vs G	0.65	0.41-1.04	0.07	1.24	0.40-3.79	0.71
IL1B (rs1143627)						
Any G vs AA	0.44	0.22-0.89	0.02	0.13	0.01-1.26	0.08
G vs A	0.58	0.36-0.91	0.02	0.42	0.14-1.27	0.13
IL6 (rs1800795)						
Any C vs GG	1.04	0.55-198	0.9	1.14	0.48-2.69	0.77
C vs G	1	0.61-1.65	0.97	1.2	0.61-2.36	0.59
Ethnicity (Caucasian)	0.71	0.30-1.66	0.43	0.69	0.22-2.14	0.52
Age	0.98	0.95-1.11	0.25	0.98	0.95-1	0.27
Male sex	0.64	0.34-1.22	0.18	0.55	0.24-1.29	0.17

Single and multivariable analysis displayed no association between the polymorphisms and hemorrhage presentation (Table 4).

**Table 4:** The association of studied polymorphisms with the clinical presentation of intracerebral hemorrhage in patients harboring brain arteriovenous malformation. OR, odds ratio calculated both in dominant and multiplicative models for the minor allele; CI, confidence interval; The single analysis calculated P values were based on 2-sided χ²-tests; * Fisher test; Logistic regression was used for multivariable analysis controlling for Caucasian ethnicity, age, male gender, and a Spetzler-Martin Grade III or higher.

	Single Analysis		Multivariable Analysis			
Risk Factor	OR	95% CI	P	OR	95% CI	P
APOE						
E2 carriers	1.38	0.21-9.01	1 *	0.54	0.05-6.32	0.63
ACVRL1 (rs2071219)						
Any G vs AA	1.69	0.55-5.26	0.36	2.46	0.44-13.94	0.31
G vs A	1.27	0.57-2.87	0.56	1.69	0.51-5.6	0.39
ENG (rs11545664)						
Any T vs CC	1.33	0.36-4.92	0.67	0.74	0.06-8.63	0.81
T vs C	1.28	0.38-4.34	0.69	0.9	0.1-8.07	0.93
TNF (rs361525)						
Any A vs GG	0.88	0.16-4.81	1 *	0.2	0.02-2.83	0.24
A vs G	0.88	0.17-4.59	1 *	0.26	0.03-2.39	0.23
IL1B (rs16944)						
Any A vs GG	0.75	0.24-2.3	0.62	1.35	0.18-9.96	0.77
A vs G	1.19	0.52-2.73	0.67	1.42	0.45-4.51	0.56
IL1B (rs1143627)						
Any G vs AA	0.76	0.45-1.71	0.3	1.45	0.27-7.46	0.71
G vs A	0.9	0.4-2	0.79	1.02	0.33-3.12	0.98
IL6 (rs1800795)						
Any C vs GG	1.08	0.36-3.24	0.89	0.49	0.07-3.26	0.46
C vs G	0.84	0.36-1.95	0.69	0.44	0.11-1.74	0.24

## Discussion

In this case-control study, we investigated the potential associations between polymorphisms in TNF (-238G>A (rs361525)), IL1B (-511C>T (rs16944)), IL1B (-31T>C (rs1143627)), ACVRL1 (IVS3-35A>G (rs2071219)), ENG (207G>A (rs11545664)), IL6 (-174G>C (rs1800795)), and APOE with the risks of developing BAVM and ICH. A trend towards an association was found between IL1B polymorphisms (rs16944 and rs1143627) and BAVM risk. These findings were not supported by a multivariable analysis. No associations were found for hemorrhagic presentation.

The association between BAVMs and well defined genetic disorders, such as hereditary hemorrhagic telangiectasia (HHT), highlights the importance of genetic contributions to pathogenesis [5]. The majority of HHT cases involve loss-of-function mutations in two genes originally implicated in transforming growth factor-beta (TGFB) signaling pathways: (1) ENG, encoding an accessory protein of TGFB receptor complexes; and (2) ACVRL1, encoding a transmembrane kinase [6].

In a study performed by Pawlikowska et al. [7], seven SNPs in ENG were analyzed in 177 BAVM patients. Two of the SNPs (rs10987759 and rs11545664) showed only a trend toward an association with BAVM in a full genotype model that did not reach statistical significance.

The same study reported that an intronic variant of ACVRL1, rs2071219, was present at a higher frequency in BAVM cases compared to healthy controls (Any A versus GG univariable logistic regression; OR = 2.47; 95% CI = 1.38–4.44; P = 0.002) [7].

APOE is an important suppressor of TNF secretion from glial cells. The APOE ε2 genotype is implicated in the pathogenesis of BAVM [4]. Longitudinal imaging studies performed on 284 BAVM patients were evaluated to determine if additional intracranial hemorrhaging occurred after diagnosis. The APOE ε2 allele (HR = 8.71; 95% CI = 1.4–53.9; P = 0.020; multivariate model adjusting for clinical presentation), but not the APOE ε4 allele, was associated with hemorrhaging in the natural course [8]. Additionally, another cohort consisting of 255 patients undergoing BAVM treatment (embolization, arteriovenous malformation resection, radiosurgery or any combination of these treatments) was genotyped and longitudinally followed (mean follow-up period of 1.9 years, and an interquartile range of 1.6 years). The authors suggested that the APOE genotype ε2 may contribute to an increased risk of a hemorrhage after BAVM treatment (HR = 3.2; 95% CI = 1.0–9.7; P = 0.042) [9].

BAVMs are sites of active inflammation. Inflammatory cytokines, including TNF and some interleukins, are potent stimulators of both angiogenesis and blood-brain barrier breakdown and could contribute to the progression and rupture of lesions [10].

The association between TNF polymorphisms (rs361525 and rs1800629) and the risk of ICH was longitudinally studied in 280 BAVM patients by Achrol et al. [11]. The rs361525 SNP was associated with an increased risk of intracranial hemorrhaging during the natural course of BAVM (HR = 4.01; 95% CI = 1.31–12.29; P = 0.015 adjusted for age, ethnicity, and history of ICH). The same authors also published another prospective study reporting an association between the rs361525 allele and ICH risk (HR = 3.5; 95% CI = 1.3–9.8; P = 0.016) in patients who underwent some type of treatment (embolization, surgical resection, radiosurgery or any combination) [9]. However, a case-control study focusing on the association between the rs1800629 and rs361525 alleles and ICH presentation did not reach significance (uncorrected P = 0.055 and P = 0.59, respectively) [4].

Patients (n = 410) with BAVM were genotyped for SNPs in the IL1B promoter (rs16944, rs1143627, and rs1143634). A survival analysis of the time to ICH was performed. Patients with the rs1143627 CC genotype (HR = 2.7; 95% CI = 1.1–6.6; P = 0.029) or the rs16944 TT genotype (HR = 2.6; 95% CI = 1.1–6.5; P = 0.039) had a greater risk of ICH. These results were adjusted for the age at diagnosis, gender, ethnicity, and hemorrhagic presentation. To determine if IL1B SNPs were associated with BAVM susceptibility, a subsequent case-control analysis restricted to Caucasians was performed. The risk of BAVM was higher in subjects with the rs1143627 CC (OR = 3.2; 95% CI = 1.7–6.1; P < 0.001), rs16944 TT (OR = 3.4; 95% CI = 1.7–6.7; P < 0.001), and rs1143634 CC (OR = 1.8; 95% CI = 1.2–2.7; P = 0.004) genotypes after adjusting for age and sex [4].

The IL1B polymorphism rs16944 was analyzed in 101 unrelated BAVM patients and in 210 healthy subjects. No significant differences were found in the distribution of either genotypic or allelic frequency between the cases and controls. A Kaplan-Meier analysis (mean of follow-up was 7.74 ± 2.58 years) revealed that BAVM patients carrying one or two copies of the T allele for rs16944 show a trend towards a higher risk of rebleeding compared to the other patients (CT + TT vs CC, χ² = 3.678, P =.055) [12].

BAVM cases and healthy controls of self-reported Latino ethnicity (n = 294) were tested for 83 ancestry informative markers. The GG genotype of the rs1800795 polymorphism for IL6 was associated with an increased risk of BAVM (OR = 1.96, 95% IC = 1.03–3.72, P = 0.039, adjusted for age, sex, and individual ancestry estimates) [13].

Another study evaluated the clinical presentation of 180 BAVM patients. The frequency of the rs1800795 GG genotype for IL6 was significantly greater in hemorrhagic patients than in patients with unruptured BAVM (OR = 2.43; CI = 1.04 to 5.68; P = 0.003 corrected for age, gender, and ethnicity). In this study, BAVM size (OR = 2.36; 95% CI = 1.12–4.96; P = 0.004) and exclusively deep venous drainage (OR = 2.36; 95% CI = 1.12–4.96; P = 0.001) were significantly associated with ICH presentation in rs1800795 GG homozygotes [4].

Several limitations of our study need to be addressed. The sample size may not have been large enough to detect small effects from SNPs of low penetrance. Case-control studies in admixed populations, like ours, are susceptible to genetic confounding. A large sample size with replicative studies and the control for genetic ancestry informative markers could overcome these limitations. False positives (type I error) for the IL1B -31T>C allele (rs1143627) in a single analysis may have occurred after multiple testing. The Bonferroni correction was not applied because significance was not maintained in the logistic regression.

It should be noted that it is important to perform genome-wide association studies (GWAS) in addition to candidate gene studies. These studies permit the analysis of the entire human genome while being unconstrained by prior hypotheses, provide important clues to genomic functional and pathophysiologic mechanisms, demonstrate gene-gene interactions, and detect high-risk haplotypes.

However, GWAS can also be problematic because the large number of statistical tests that are performed can lead to false-positive results (spurious association) requiring the replication of the findings [14] or false-negative results (Bonferroni overcorrection). Furthermore, because BAVM is a rare disease, a significant international collaboration is needed to assemble a cohort large enough to study the genetics of BAVM and the risks of rupture.

## Conclusions

In our sample from a south Brazil population, we found no association between the risks of BAVM and ICH presentation with the following selected polymorphisms: TNF -238G>A (rs361525), IL1B -511C>T (rs16944), IL1B -31T>C (rs1143627), ACVRL1 IVS3-35A>G (rs2071219), ENG 207G>A (rs11545664), IL6 -174G>C (rs1800795), and APOE. The role of these polymorphisms in the pathogenesis of BAVM and the risk of rupture warrants larger replicative studies with an ethnically diverse population, including large GWAS.
